# Technical development of transcutaneous electrical nerve inhibition using medium-frequency alternating current

**DOI:** 10.1186/s12984-018-0421-8

**Published:** 2018-08-20

**Authors:** Yushin Kim, Hang-Jun Cho, Hyung-Soon Park

**Affiliations:** 1 Major in Sport, Health & Rehabilitation, Department of Health Administration and Healthcare, Cheongju University, Cheongju, 28503 Republic of Korea; 2 Department of Mechanical Engineering, Korea Advanced Institute of Science and Technology (KAIST), Daejeon, 34141 Republic of Korea

**Keywords:** Electrical stimulation, Nerve inhibition, Kilohertz-frequency alternating current, Surface electrode, Force, Sensory, Pain, Motor

## Abstract

**Background:**

Innovative technical approaches to controlling undesired sensory and motor activity, such as hyperalgesia or spasticity, may contribute to rehabilitation techniques for improving neural plasticity in patients with neurologic disorders. To date, transcutaneous electrical stimulation has used low frequency pulsed currents for sensory inhibition and muscle activation. Yet, few studies have attempted to achieve motor nerve inhibition using transcutaneous electrical stimulation. This study aimed to develop a technique for transcutaneous electrical nerve inhibition (TENI) using medium-frequency alternating current (MFAC) to suppress both sensory and motor nerve activity in humans.

**Methods:**

Surface electrodes were affixed to the skin of eight young adults to stimulate the median nerve. Stimulation intensity was increased up to 50% and 100% of the pain threshold. To identify changes in sensory perception by transcutaneous MFAC (tMFAC) stimulation, we examined tactile and pressure pain thresholds in the index and middle fingers before and after stimulation at 10 kHz. To demonstrate the effect of tMFAC stimulation on motor inhibition, stimulation was applied while participants produced flexion forces with the index and middle fingers at target forces (50% and 90% of MVC, maximum voluntary contraction).

**Results:**

tMFAC stimulation intensity significantly increased tactile and pressure pain thresholds, indicating decreased sensory perception. During the force production task, tMFAC stimulation with the maximum intensity immediately reduced finger forces by ~ 40%. Finger forces recovered immediately after stimulation cessation. The effect on motor inhibition was greater with the higher target force (90% MVC) than with the lower target (50% MVC). Also, higher tMFAC stimulation intensity provided a greater inhibition effect on both sensory and motor nerve activity.

**Conclusion:**

We found that tMFAC stimulation immediately inhibits sensory and motor activity. This pre-clinical study demonstrates a novel technique for TENI using MFAC stimulation and showed that it can effectively inhibit both sensory perception and motor activity. The proposed technique can be combined with existing rehabilitation devices (e.g., a robotic exoskeleton) to inhibit undesired sensorimotor activities and to accelerate recovery after neurologic injury.

## Background

In patients with neurologic disorders, the presence of undesired sensorimotor activity is a major clinical challenge. For example, hypersensitization, spasticity, hypertonia, and/or dystonia causes sensory and motor impairment in patients with brain injuries. To control pathologic neuromuscular conditions, researchers have introduced various therapeutic interventions such as transcutaneous electrical stimulation, oral medications, injections of botulinum neurotoxin, local anesthetics, physical therapy, rehabilitation robotic training, and surgery [1–3].

Compared to analgesic drugs, transcutaneous electrical stimulation has fewer side effects and, thus, has become a popular therapeutic technique for inhibition of undesired sensory activity, such as excessive pain or hyperalgesia [4]. For instance, to suppress excessive pain, surface electrodes are attached at the site of pain and a low frequency pulsed current (1–100 Hz) is applied [5]. Low frequency currents have also been used in functional electrical stimulation (FES) or neuro-muscular electrical stimulation (NMES), in which the current acts an excitatory agent for muscle contraction [6]. Similarly, current techniques for transcutaneous electrical stimulation have been developed using low frequency currents aimed at either inhibiting sensory perception or exciting muscle fibers (i.e., inducing contraction). This technique cannot be used for motor inhibition, which is essential for suppression of undesired motor activities (e.g., spasticity, hypertonia, dystonia).

There is some evidence supporting the feasibility of using transcutaneous electrical nerve inhibition (TENI) to reduce undesired nerve activity [7, 8]. Previous animal studies demonstrated that electrical stimulation with medium-frequency alternating currents (MFAC, 2 kHz – 40 kHz) inhibits motor nerve activity [8–12]. Those studies found that MFAC inhibits peripheral nerve activity and muscle force production when implanted electrodes directly deliver electrical currents to the peripheral nerve [9, 12]. Furthermore, the MFAC stimulus has shown intensity-dependent characteristics (i.e., a higher intensity stimulus induces greater nerve inhibition) and time-dependent characteristics (i.e., suppression occurs immediately after stimulus application) [8–11]. Most literature reporting MFAC techniques has been limited to animal studies because current MFAC techniques require surgical procedures to directly implant or insert electrodes into the target muscle. Development of a technique in which MFAC could be applied using transcutaneous electrical stimulation, with good skin penetration and effective nerve inhibition (e.g., TENI), could allow wider clinical application to suppress undesired sensory and motor activities. In addition, future applications of TENI could include combinations with other rehabilitation engineering techniques, such as with a robotic exoskeleton, to reduce pain and/or spasticity and to enhance functional recovery in neurologic patients.

One potential challenge with TENI is the proper targeting of axon fibers in a particular peripheral nerve. In general, the target of electrical stimulation, a peripheral nerve, is located below a layer of fat and muscle that may act as an electrical insulator [13]. Furthermore, applying MFAC to the muscle belly can induce muscle contraction rather than nerve inhibition. A previous study has proposed that MFAC delivered to the neuromuscular junction acts to release neurotransmitters at the end of the intramuscular axons [11]. This action can be avoided by moving/placing electrodes away from muscle bellies. Fortunately, there are specific regions in which peripheral nerves pass below a thin layer of subcutaneous fat and non-muscular tissue, such as the location of the median nerve proximal to the wrist. Moreover, previous studies have demonstrated that MFAC can penetrate soft tissues approximately 2.5 cm from the surface of the skin [14, 15] and may transmit a stimulating current from the skin to a layer below subcutaneous fat [16]. These results support the technical feasibility of TENI using MFAC.

In this study, we aimed to develop a technique for inhibiting human sensorimotor activities with TENI using MFAC. We hypothesized that transcutaneous MFAC (tMFAC) stimulation of the distal median nerve would 1) reduce sensory perception in the index and middle fingers and 2) inhibit force production by the two fingers. We also expected that a higher stimulus intensity would result in greater inhibition of both sensory and motor nerve activity compared to a lower stimulus intensity.

## Methods

### Design

We applied tMFAC stimulation to the distal median nerve. To confirm the effect of tMFAC stimulation on sensory perception, we performed the Semmes–Weinstein monofilament examination and pressure algometry to the index and middle fingers. To identify the motor inhibitory effects caused by TENI, we applied tMFAC stimulation for 5 s while participants continuously pressed force sensors with the index and middle fingers and measured the reduction in force during stimulation. We also monitored the safety of using TENI with MFAC.

### Participants

Eight healthy young adults (age: 24.8 ± 3.0 y, height: 172.4 ± 7.2 cm, weight: 64.9 ± 7.6 kg, six males, two females) participated in this study. All participants, except one, were right-handed. Potential participants were excluded from the study if they reported musculoskeletal pain, diabetes mellitus, hypertension, autoimmune disease, and any surgical history or neurologic disorder. Written informed consent was obtained from all participants prior to participation. The experimental protocol was approved by Institutional Review Board of the Korea Advanced Institute of Science and Technology.

### Apparatus

**Fig. 1:**
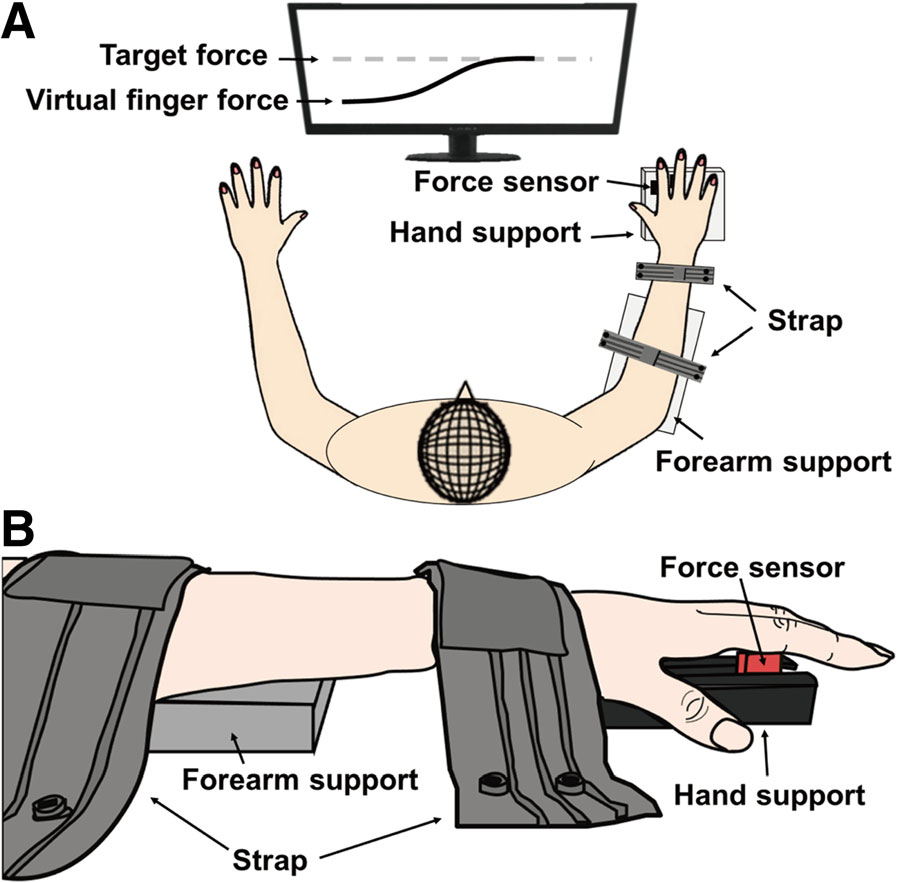
Experimental setup from top (**a**) and side views (**b**)

The participants were seated on a chair and their arms were positioned on a testing table. The height of the chair was adjusted such that participants could put their arms on the table with both shoulders at approximately 35° of abduction and 45° of flexion and elbows at approximately 45° of flexion (Fig. 1a). A rigid Styrofoam™ board was used to support both wrists and forearms.

To measure the forces from the index and middle fingers of each participant’s non-dominant hand, two piezoelectric force sensors (CSBA-20LS, Curiotech, Korea) were mounted inside a plastic frame. The position of the sensors could be adjusted in the medial–lateral direction, within a range of 100 mm, such that the sensors were placed at the head of the proximal phalanx of each participant’s index and middle fingers, these positions were maintained throughout the experiment.

Analog output signals from the sensors were processed using separate AC/DC conditioners (RW-ST01A, SMOWO, Shanghai China). A cotton cover was attached to the upper surface of each sensor to prevent friction generated by slipping and to limit the influence of finger skin temperature on the piezo-electric signals. A 16-bit A/D board (NI 6211, National Instruments, Austin, TX USA) converted processed analog input into digital signals at 1000 Hz. Data were low-pass filtered with a 3rd order Butterworth filter at 25 Hz. Raw data were acquired using LabVIEW (LabVIEW 2010, National Instruments, Austin, TX USA).

To apply tMFAC stimulation through surface electrodes (Hypoallergenic Electrodes, Roscoe Medical/Compass Health Brands, Middleburg Heights, OH USA), we used an electrotherapy device (InTENSity Select Combo II, Roscoe Medical/Compass Health Brands, Middleburg Heights, OH USA). In order to create a biphasic, steady, unmodulated alternating current of 10 kHz in a square-wave pulse, we selected the manual IF (interferential) program mode provided by the device. Two electrodes (Channel 1) were attached on the skin. The other two electrodes (Channel 2) were not used to avoid any affect due to interference. An oscilloscope (TDS2012C, Tektronix, Beaverton, OR, USA) was used to confirm the pulse (unmodulated square-wave at 10 kHz) and current intensity (mA) created at the Channel 1 electrodes. When electrical stimulation started, the stimulus intensity gradually increased for 0.3 s.

### Procedure

#### Preparation

**Fig. 2:**
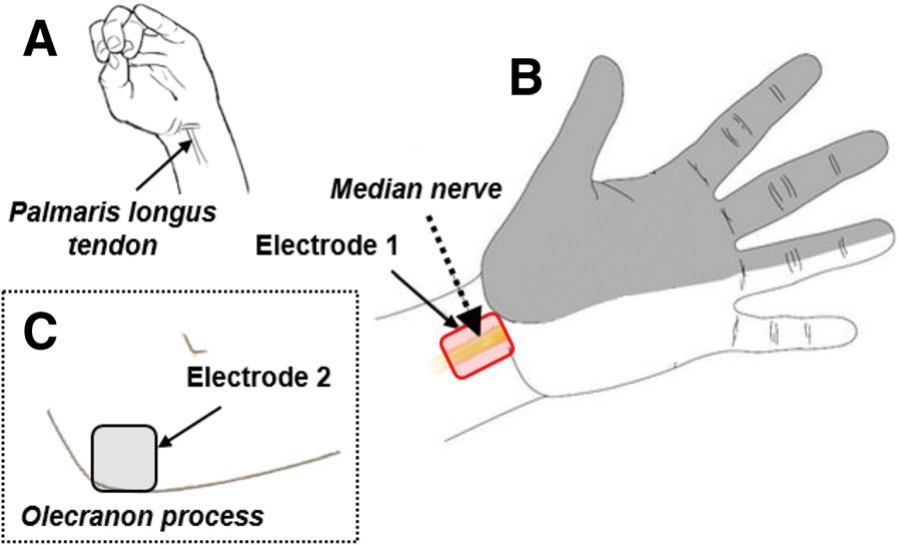
The placement of electrodes. **a** Finger-to-thumb opposition task. Participants were asked to oppose the index, middle, and ring fingers of the non-dominant hand along with wrist flexion/extension to aid in identification of the palmaris longus tendon. **b** The placement of the anode (Electrode 1) overlying the median nerve (yellow box). Shading indicates the sensorimotor distribution of the median nerve. **c** The placement of the cathode (Electrode 2) over the ipsilateral olecranon process

To identify the location of the median nerve, participants were asked to execute a finger-to-thumb opposition task with wrist flexion (Fig. 2a). Then, the locations of the palmaris longus tendon was confirmed and its radial side was used to identify the location of the median nerve. The skin over the median nerve was cleaned with a 70% isopropyl alcohol pad. Electrode 1 (2 × 1 cm) was placed on the skin overlying the median nerve near the transverse carpal ligament (Fig. 2b). Electrode 2 (5 × 5 cm) was placed over the ipsilateral olecranon process, proximal to Electrode 1 (Fig. 2c). In each test, surface electrodes were optimally placed where the sensation evoked by electrical stimulation was strongest at the tip of the index and middle fingers. Electrode placement was slightly adjusted if MFAC stimulation induced undesired muscle contraction secondary to direct stimulation of the neuromuscular junction or asynchronous firing of the nerve [11]. For example, if stimulation caused thenar muscle contraction, the location of the electrodes was moved slightly (approximately 0.5 cm) toward the ulnar or the proximal side. Subsequently, we monitored whether subjects perceived sensory changes in the median nerve-innervated areas, primarily the 2nd and the 3rd fingers but not in areas innervated by other nerves such as the 4th and the 5th fingers. To determine the maximum intensity acceptable to a participant, electrical stimulation was gradually increased to individual pain threshold [17, 18], which was found to be 31.4 ± 4.4 mA.

#### Identification of sensory inhibition

To identify the effect of tMFAC stimulation on sensory perception, we performed the Semmes–Weinstein monofilament examination and pressure algometry that measure tactile and pressure pain thresholds, respectively. Two measurements were performed to the skin at the tip of the index or middle finger. During sensory testing, the non-dominant hand was placed on a desk with the palm up. The sensory test was performed under three conditions: baseline, 100% intensity, and 50% intensity. In the baseline condition, sensory testing was performed without any stimulation. In the 100% and 50% intensity conditions, measurement was performed while applying tMFAC. In each condition, three measurement trials were conducted for each finger. Tactile and pressure pain thresholds were taken as the average of three measurements.

For the Semmes–Weinstein monofilament examination, we used a set of 20 nylon monofilaments (Touch Test Sensory Evaluators, North Coast Medical, Gilroy, CA USA), graded according to monofilament diameter. Measurements were made by pressing each monofilament to the skin. Measurements started with the smallest diameter monofilament (an ascending method of threshold testing) [19]. The monofilament was held in contact with the skin until it bent, and then removed after 1 s. Participants were asked to close their eyes and to indicate whether they could sense the monofilament stimulation [20]. In the 100% and 50% intensity conditions, we applied tMFAC for 5 s and performed monofilament examination within 1 to 3 s after starting the tMFAC stimulation, so that participants could not anticipate the onset of pressure. We recorded tactile threshold in milligram force as directed by the manufacturer and force values were presented using a logarithmic scale [21, 22].

To perform pressure algometry, we used a 1-cm diameter algometer (EFFEGI FPK 20, Facchini SRL, Alfonsine RA Italy). Pressure was applied to the skin in a perpendicular direction using the algometer. Participants were instructed to report when they felt a transition from a touch or pressure sensation to noxious pain, corresponding to each individual’s pressure pain threshold. Pressure was increased at a rate of 1 kg/cm^2^ and released after the subject reported pain [23]. This method has previously shown high trial-to-trial reliability [24]. In the 100% and 50% intensity conditions, we applied pressure after starting the tMFAC stimulation. When pressure pain threshold was identified, the tMFC stimulation was stopped. All pressure pain threshold values were recorded in kg/cm^2^.

#### Identification of motor inhibition

To measure finger forces, a customized plastic frame (120 × 110 mm) with an arch was placed underneath the palm to maintain approximately 0° of wrist extension and metacarpophalangeal flexion (Fig. 1b). Two straps fixed the participant’s wrist and forearm to a testing platform to limit force transmission from proximal muscles and from the elbow and shoulder joints.

A finger-pressing task was designed, such that subjects pressed a force sensor with the head of the proximal phalanx of each index and middle finger (Fig. 1b). Wrist and hand position was optimized to maximize the contribution of the intrinsic hand muscles (e.g., lumbrical and interosseous muscles) [25, 26]. To determine the target force, participants were asked to press the sensors using maximum voluntary contraction (MVC), such that they produced their maximum finger force. During MVC measurement, a digital monitor provided visual feedback on virtual finger forces, calculated as the sum of the forces produced by the index and middle fingers. MVC measurement were repeated three times and the values were averaged.

After three to five practice trials, the finger-pressing tasks were conducted using electrical stimulation. The finger-pressing tasks were conducted under four conditions with two target forces (90% and 50% MVC) and two tMFAC stimulation intensities (100% and 50% maximum intensity). Each of the four conditions were repeated three times consecutively. In total, 12 experimental trials were conducted with a 60-s rest period between each trial.

In the finger-pressing tasks, participants were asked to match the virtual finger force to the target force. A digital monitor displayed two lines corresponding to the target force and the participant’s virtual finger force. The finger-pressing task was performed for 15 s. The two target forces, 90% and 50% of MVC, were selected in random order. Raw force values were recorded and displayed in newtons (N).

During the finger-pressing tasks, we applied tMFAC stimulation within 1 to 5 s after starting the task, such that participants could not anticipate the onset of stimulation. To avoid a startle response associated with the onset of electrical stimulation, the stimulus intensity gradually increased for 0.3 s after the time of onset. The stimulus immediately stopped after 5 s. Participants were asked to continue performing the finger-pressing task whether or not stimulation sensations were felt. Visual feedback on force production was maintained during electrical stimulation.

The participants were also asked to report any paresthesias, dysesthesias, or fatigue. If hypersensitive fear, severe fatigue, discomfort, or any abnormal change was reported during a task trial, the experiment was immediately stopped.

### Data analysis

**Fig. 3:**
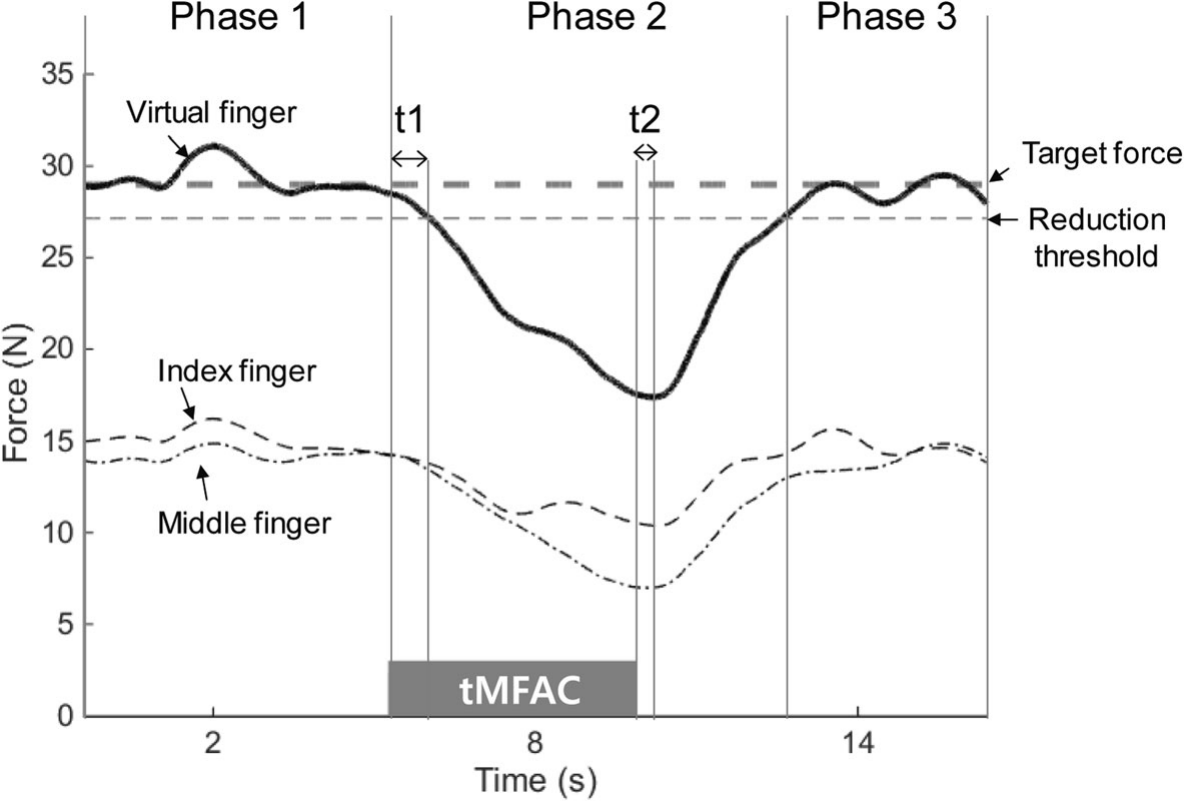
A sample of a force trajectory in the finger-pressing task using the index and middle fingers. Virtual finger forces represent the sum of the index and middle finger forces. The target force is determined using 90% and 50% of a maximum voluntary contraction in the virtual finger. The reduction threshold is a reference value calculated by subtracting three standard deviations from the mean force during Phase 1. Phase 1 is the baseline period wherein the participants matched the virtual finger force to the target force before transcutaneous medium-frequency alternating current (tMFAC) stimulation was delivered. Phase 2 is the period from onset of tMFAC stimulation to the point that the virtual finger force recovers to reach the reduction threshold. Phase 3 is the period in which virtual finger force has completely recovered. In Phase 2, t1 and t2 were defined as onset and offset times for blockade of motor neuron signals, respectively. For instance, t1 represents the time from tMFAC stimulation onset to the time when virtual finger force falls below the reduction threshold. Also, t2 represents the time from tMFAC stimulation cessation to the time when virtual finger force begins to increase

All finger forces were normalized by each participant’s virtual finger force at MVC. Data are presented as a percentage of MVC (% MVC). To investigate changes in force production induced by tMFAC stimulation, we divided the time-varying force trajectory measured during the finger-pressing task into three phases, based on virtual finger force values (Fig. 3). Phase 1 was the baseline period in which the participants successfully matched the virtual finger force with the target force, prior to the stimulation. To determine a reference value for meaningful force changes, we calculated a reduction threshold based on the 68–95–99.7 rule, in which the values are skewed if the values in a normally distributed data set are less than two standard deviations from the mean [27]. A reduction threshold was calculated [Reduction Threshold = Mean Total Finger Force – 2 x (Standard Deviation of the Total Finger Force)] using the virtual finger force for the 1 s prior to the onset of stimulation [27, 28]. Phase 2 was the period that MFAC influenced finger force production. In this period, t1 and t2 were defined as onset and offset times for inhibitory effects on motor neuron signals. Specifically, t1 was defined as the time interval in which finger forces decreased below the reduction threshold, after introduction of tMFAC stimulation and t2 was defined as the time interval between cessation of the stimulation to the time of minimum force production, indicating the time for recovery of finger force production from MFAC effects. Finally, Phase 3 was defined as the period after the stimulation, in which finger forces completely recovered above the reduction threshold.

To examine finger-pressing performance during the task, the mean squared error (MSE) of two-finger forces with respect to the target force was calculated for each phase. MSE values were computed using raw force data (N) to allow comparison of finger-pressing performance between different target force conditions (e.g., 90% and 50% MVC). To quantify force reduction induced by tMFAC stimulation, the mean and minimum values of the index, middle, and two-finger forces in each phase were calculated using the normalized force data (% MVC). Data collected from the same experimental conditions were averaged for each participant.

### Statistical analysis

Data are presented using mean and standard deviation values. To compare tactile thresholds among baseline, 100%, and 50% intensities of tMFAC stimulation, the Friedman test was used for each of the index and middle fingers, and then as a post-hoc test. One-way repeated measures analysis of variance (ANOVA) was performed for the comparison of pressure pain thresholds among baseline, 100%, and 50% intensities of tMFAC stimulation in the index and middle fingers. If ANOVA results indicated significant interactions, multiple pairwise comparisons were conducted using Duncan’s new multiple range test. The Wilcoxon signed-rank test was used with a Bonferroni adjustment (accepted α was 0.0167). One-way repeated measures ANOVA was also conducted to compare finger forces between the three phases using normalized mean and minimum values. To compare MSE values with three factors, the three-way repeated measures ANOVA was conducted (i.e., target forces (90% vs. 50%), stimulation intensities (100% vs. 50%), and phases (Phase 1 vs. 2 vs. 3)). The intraclass correlation coefficient (ICC) was calculated for trial-to-trial reliability of pain threshold and MVC measurements. In the study, the level of significance was set at *p* < 0.05.

## Results

In the monofilament test, tMFAC stimulation increased tactile thresholds in both the index and middle fingers (Table 1). Also, higher stimulation intensity provided a greater inhibition of tactile sensation. Stimulation at 100% intensity significantly increased the tactile threshold to approximately twice that of the baseline condition. Stimulation at 50% intensity also significantly increased the tactile threshold to approximately 1.5 times the baseline condition. Stimulation at either 100% or 50% intensity also significantly increased the pain threshold (Table 1). Similar to the tactile threshold, a higher intensity created a greater inhibitory effect on pain. On average, the pain threshold increased 43% and 17% over baseline levels with 100% and 50% stimulation intensities, respectively. The trial-to-trial reliability was good for pressure pain thresholds (ICC ≥ 0.778).

**Table 1:** Changes in tactile [log10 (10 × force in mg)] and pain threshold (kg/cm^2^) after tMFAC stimulation

	Baseline	100% intensity	50% intensity	Significance
Index finger				
Tactile threshold^a)^	2.3 (2.3–2.6)	4.7 (4.3–6.0)	3.6 (3.1–3.6)	χ^2^: 16.00, *p*: < 0.001
Pain threshold^b)^	3.2 ± 0.4	4.4 ± 0.5	3.7 ± 0.2	F: 20.042, *p*: < 0.001
Middle finger				
Tactile threshold^a)^	2.5 (2.3–2.6)	5.0 (4.7–6.0)	3.9 (3.4–4.3)	χ^2^: 16.00, *p*: < 0.001
Pain threshold^b)^	3.0 ± 0.3	4.5 ± 0.5	3.6 ± 0.3	F: 28.230, *p*: < 0.001

Values are expressed as median values (inter-quartile range)^a)^ and means ± standard deviations^b)^

The mean virtual MVC was 28.8 ± 2.9 N, contributed by the index (15.1 ± 1.6 N) and middle (13.7 ± 1.8 N) fingers. The trial-to-trial reliability was good for MVC measurement (ICC = 0.737). The target force in the finger-pressing task was 25.8 ± 2.5 N at a level of 90% MVC and 14.9 ± 2.1 N at 50% MVC.

**Fig. 4:**
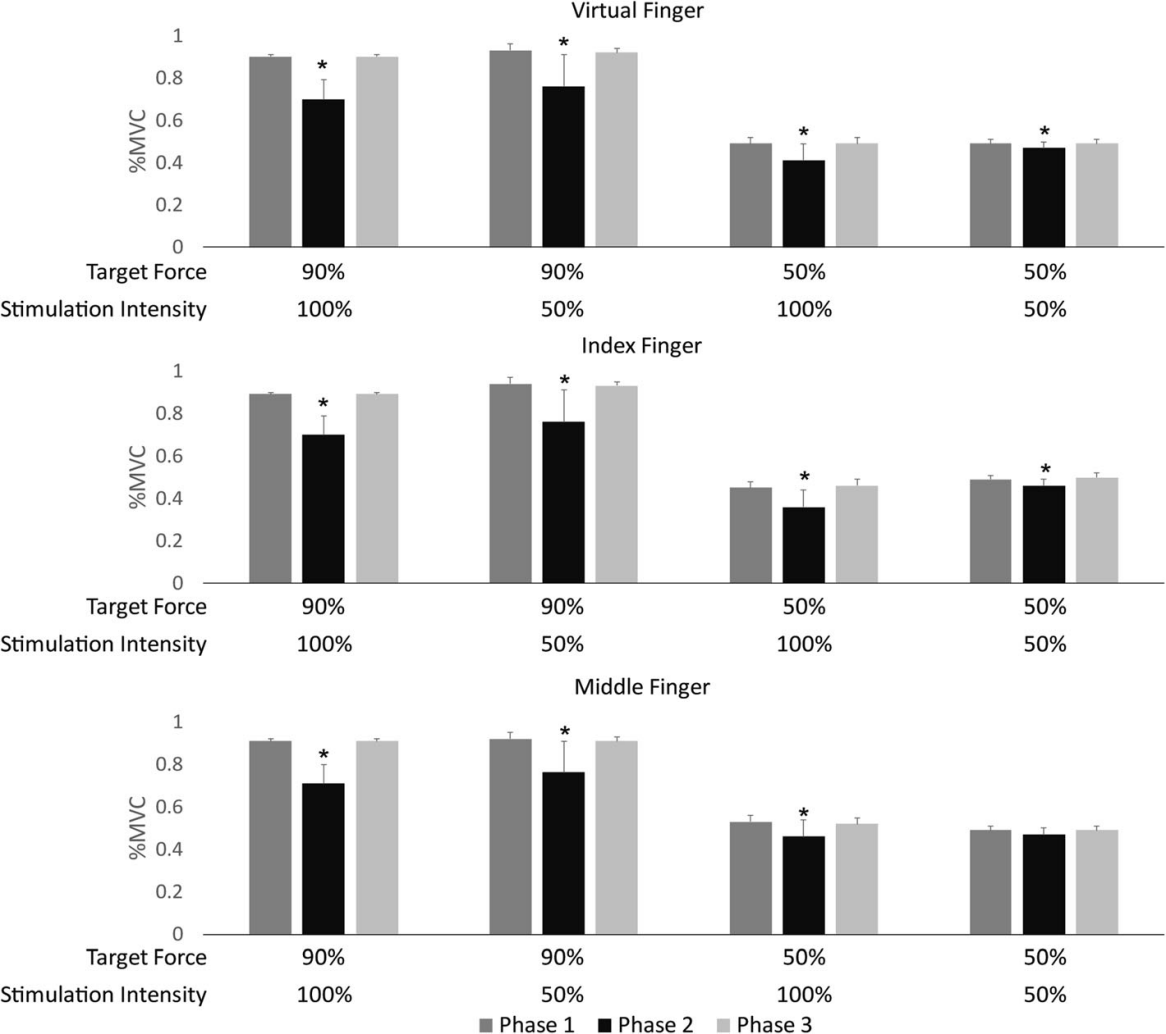
Mean finger force by phase in each experimental condition. Data are presented as mean ± standard deviation of force, normalized by the maximal voluntary contraction (%MVC) of corresponding fingers for each participant. Virtual finger force indicates the sum of the index and middle finger forces. *: significantly lower force in Phase 2 than that of Phases 1 or 3 (*p* < 0.05, Duncan’s new multiple range test)

**Fig. 5:**
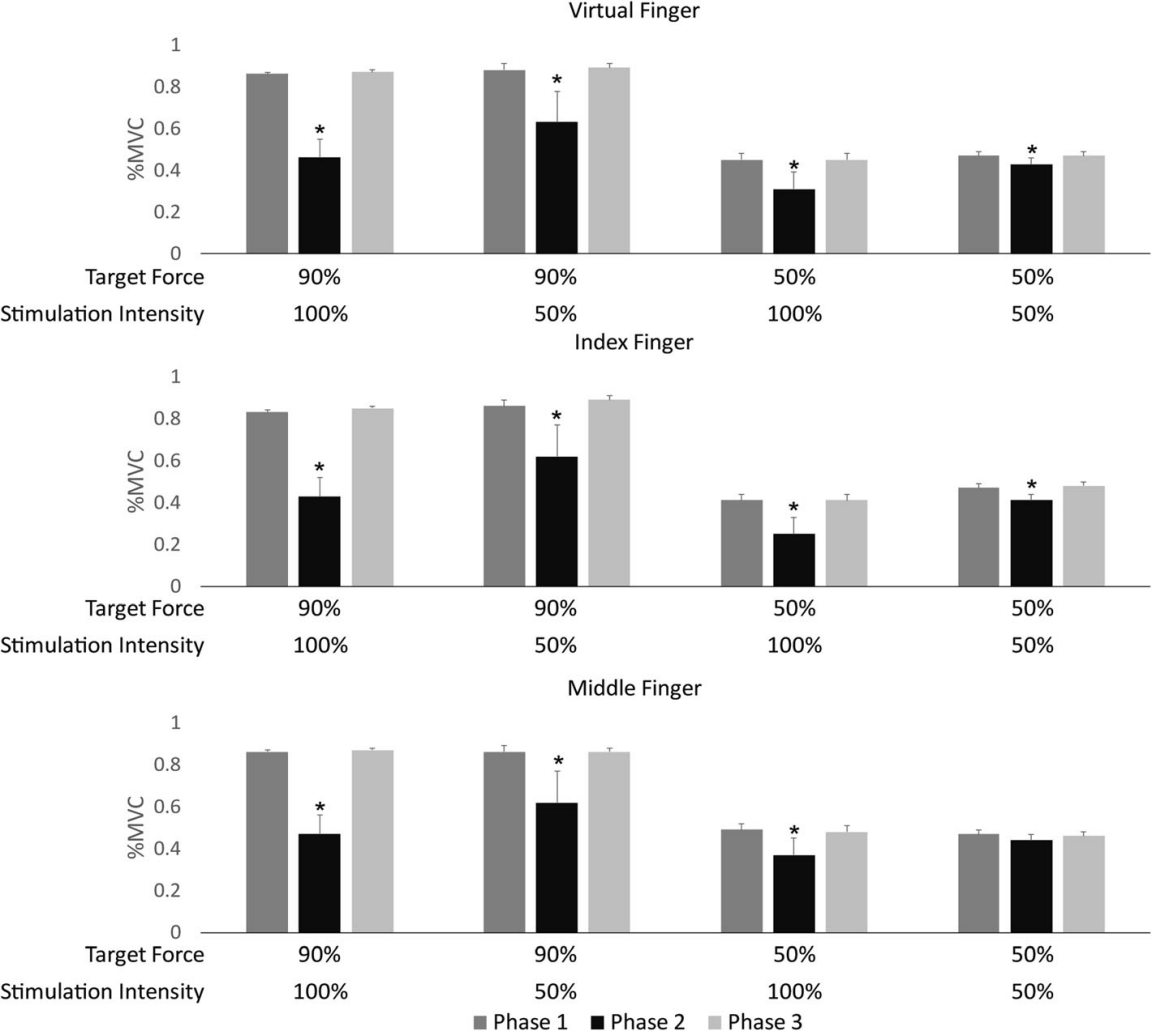
Minimum finger force by phase in each experimental condition. Data are presented as mean ± standard deviation of force, normalized by the maximal voluntary contraction (%MVC) of corresponding fingers for each participant. Virtual finger force indicates the sum of the index and middle finger forces. *: significantly lower force in Phase 2 than that of Phases 1 or 3 (*p* < 0.05, Duncan’s new multiple range test)

tMFAC stimulation significantly reduced mean finger forces during Phase 2 when compared to Phases 1 and 3 in most experimental conditions (Fig. 4). Specifically, significant differences in the mean and minimum forces for all fingers (index, middle, and virtual fingers) were seen between the three phases, with the single exception of the experimental condition with 50% MVC and 50% stimulation intensity, in which there was no difference in the mean and minimum forces generated by the middle finger between phases. Changes in the minimum forces in each phase support the finding that tMFAC stimulation reduced the finger forces by almost half, especially in the 90% MVC and 100% stimulation intensity experimental condition (Fig. 5). In the minimum forces, tMFAC stimulation at maximum intensity decreased the virtual finger force by 40% and 14% when the target force was 90% and 50% MVC, respectively. When the intensity was 50% of pain threshold, the virtual finger force was reduced by 25% and 4% at target forces of 90% and 50% MVC, respectively.

**Fig. 6:**
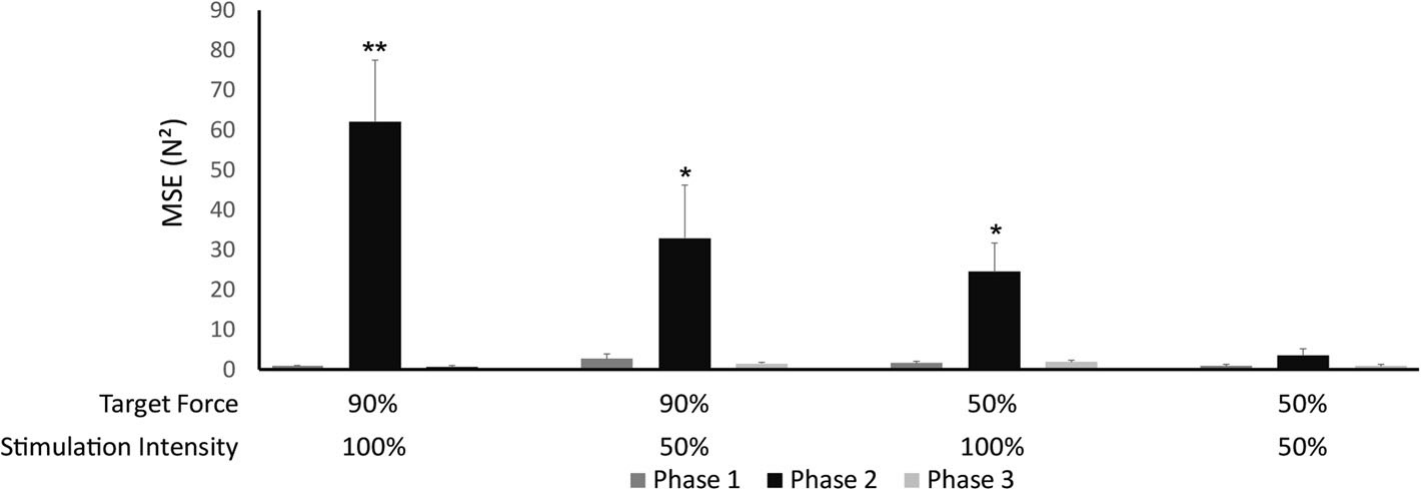
Comparison of mean squared error (MSE) between experimental conditions. MSE was compared with three factors, i.e., target forces (90% vs. 50%), stimulation intensities (100% vs. 50%), and phases (Phase 1 vs. 2 vs. 3). Phases 1, 2, and 3 indicate pre-stimulation, stimulation, and post-stimulation periods, respectively. Here, a higher MSE value represents performance error that finger forces are produced apart from a target during a finger-pressing task. **: The condition presenting the highest MSE value compared to the other conditions (*p* < 0.05, Duncan’s new multiple range test). *: The condition presenting the second highest MSE value in experimental conditions (*p* < 0.05, Duncan’s new multiple range test)

Stimulation with the tMFAC technique altered motor performance when the participants performed a finger-pressing task (Fig. 6). A significant difference between MSE values was shown between target forces (90% vs. 50%, F = 6.987, *p* = 0.033), stimulation intensities (100% vs. 50%, F = 7.035, *p* = 0.033), and phases (Phase 1 vs. 2 vs. 3, F = 8.291, *p* = 0.004). Significant interactions were found between factors (*p* < 0.05). Three groups were identified by posthoc testing: (1) The experimental condition with 90% MVC and 100% stimulation intensity demonstrated the highest MSE during Phase 2 (*p* < 0.05; noted ** in Fig. 6), with respect to the other experimental conditions. (2) Two other experimental conditions, 50% MVC with 100% stimulation intensity and 90% MVC with 50% stimulation intensity, demonstrated elevated MSE during Phase 2, compared to the other experimental conditions (*p* < 0.05; noted * in Fig. 6). (3) MSE values during all other experimental conditions were not significantly different.

The time intervals t1 and t2, were tested to determine onset and offset times of inhibition and recovery of motor responses by tMFAC stimulation. In general, t1 values showed that motor inhibition occurred within one second of stimulation onset. Inhibition and recovery responses under sub-maximal stimulation intensity were usually faster when compared to those at maximum stimulation intensity. When the target force was 90% MVC, the inhibition period (t1) was 0.61 ± 0.19 s for 100% stimulation intensity and 0.38 ± 0.31 s for that of 50% intensity and the recovery period (t2) was 0.34 ± 0.25 s at 100% intensity and 0.19 ± 0.16 s at 50% intensity. When the target force was 50% MVC, t1 was 0.58 ± 0.25 s for 100% intensity and 0.42 ± 0.24 s for 50% intensity, while t2 was 0.17 ± 0.14 s and 0.21 ± 0.18 s for 100% and 50% stimulation intensity, respectively.

No participants dropped out of the experiment and no adverse effects were reported by the participants during or after the stimulation.

## Discussion

The current study demonstrates that tMFAC inhibits both sensory and motor nerve activity. To immediately suppress human sensory perception, various technical approaches for transcutaneous electrical stimulation have been suggested in both low- and medium-frequency ranges [5, 8]. However, the transcutaneous electrical stimulation that immediately depresses human motor activities has not been investigated. Only earlier animal studies using implanted electrodes have demonstrated that delivering MFAC to the peripheral nerve can immediately depress motor nerve conduction, resulting in muscle force reduction [10–12]. Thus, it remained unclear whether tMFAC applied through surface electrodes can inhibit both sensory and motor nerve activity. To our knowledge, this is the first human behavior study to describe a TENI technique that reduces both sensory perception and muscle force production by applying tMFAC. We found that tMFAC stimulation immediately reduced finger force production and demonstrated recovery from the inhibition effect after cessation of stimulation. We have also shown that inhibition effects by tMFAC were safe in our healthy participants.

The inhibition effect of tMFAC differed with the level of stimulation intensity. Our results showed that tMFAC stimulation with a higher intensity created a stronger inhibition effect in nerve responses. Similar results were also found in earlier animal studies that applied MFAC to the peripheral nerve through implanted electrodes [9–12]. In human subjects, one study demonstrated that MFAC delivered at fixed stimulation intensity using implanted electrodes reduced pain in amputees [29]. Most previous studies using non-invasive methods applied electrical stimulation above the muscle belly [14, 16, 30] and used modulated tMFAC, e.g., interferential current [14, 16]. Those studies focused on delivering interferential current below the muscle belly for sensory inhibition only. Also, kilohertz carrier frequency was used to deliver interferential currents (50–100 Hz) deeper under the tissue. In a very relevant study, Avedano-Coy et al. compared the sensory inhibition effect between tMFAC and TENS applied to the radial nerve in the forearm [7]. They showed that pressure pain thresholds increased about 20% with tMFAC, whereas TENS increased the thresholds by 30%. Although TENS was superior to tMFAC in [7], our result showed a greater increase in pressure pain threshold with tMFAC (43% increase). The major reason would be that we applied greater current intensity. Given that the stimulus intensity of tMFAC (18.0 ± 3.5 mA) used in [7] was a motor threshold that was about 57% of the pain threshold (31.4 ± 4.4 mA) used in the present study, our result (43% increase) is consistent with the result in [7]. Furthermore, it should be noted that the greater current intensity (31.4 ± 4.4 mA) used for tMFAC in our study compared to that used with TENS (16.6 ± 4.0 mA) in [7] does not mean that greater electrical energy was delivered to the subjects, because the magnitude of voltage decreases as frequency increases due to the frequency-dependent characteristics of skin impedance [31]. However, as applying MFAC at low intensity is insufficient to show nerve inhibition effects [11], further studies are necessary to demonstrate the efficacy of MFAC and TENS at various intensities.

In this study, we identified finger force reduction caused by tMFAC, although the intensity of the current was above the motor threshold. Low-frequency currents (1–100 Hz) used for FES or NMES increase muscle force when the intensity of the current is increased above the motor threshold. However, this is not the case with the MFAC. It has been demonstrated that the application of MFAC causes a change in membrane potential that is localized to the area just under the electrodes [32, 33] and is not transmitted to the neuromuscular junction [34]. However, a few cases reported in the literature describe MFAC-induced muscle contraction [11]. The first case is that of transient firing of a nerve at the onset of MFAC stimulation resulting in a reflexive muscle response. This response is inevitable, but does not last longer than 2 s. Thus, we applied electrical stimulation for 5 s and measured steady state responses. The second case is that of MFAC directly delivered to the neuromuscular junction where it acts to release neurotransmitters at the end of the intramuscular axons. Therefore, we positioned surface electrodes away from the muscle belly. The third case is that of asynchronous firing observed when nerve responses are not synchronized with the stimulating pulses. In a previous study [11], asynchronous firing was observed if electrical intensity was not adequate to completely stimulate the nerve, and an increase in stimulation intensity could eliminate asynchronous firing. Thus, we increased stimulation intensities up to the pain threshold. The median nerve consists of both sensory and motor fibers. Thus, it is reasonable to infer that tMFAC stimulation depolarizes the membrane potential in both the sensory and the motor fibers, resulting in the inhibition of sensory perception and finger force production.

We also found that higher intensity resulted in a stronger inhibition effect in finger force production. In the finger-pressing task, tMFAC stimulation at maximum intensity resulted in 40% and 14% virtual finger force reduction when the target force was 90% and 50% MVC, respectively. Since previous animal studies that used implanted electrodes showed 100% blockade of muscle force production [9–12], force reduction in this study may be attributed to a partial blockade effect. Applying higher intensity would increase the amount of force reduction but this is inapplicable in humans due to pain perception. The magnitude of decreased force output is comparable to that in a previous study in which the administration of local anesthetic agents to the distal median nerve decreased pinch grip force by 60% [35].

In this study, it is likely that tMFAC stimulation results in decreased force production through a combination of both disturbed sensory perception and suppressed peripheral nerve activity. In previous studies, digital anesthesia (that only blocks sensory feedback) reduced finger force production by 26–30% [36, 37]. Given that sensory feedback provides a net facilitatory effect on motor output in the central nervous system [38, 39], a disturbance in sensory perception produced by tMFAC stimulation may suppress motor neuron excitability [40]. In addition, we postulate that tMFAC stimulation directly inhibits motor nerve activity by inducing nerve signal propagation failure and/or neurotransmitter depletion at the neuromuscular junction [8]. In the current study, participants were instructed to press force sensors using the head of proximal phalanx with simultaneous extension of the interphalangeal joints, because this position emphasizes the contribution of intrinsic hand muscles (85%) [25]. If tMFAC stimulation affects the activity of the median nerve, the actions of lumbrical muscles innervated by the median nerve would be inhibited, resulting in a decrease in MCP flexion forces. A previous cadaver study demonstrated that during free movement of the fingers, the first lumbrical muscle acting alone can produce MCP flexion forces as low as 5 N [26]. Another study also demonstrated that the first lumbrical muscle can contribute 19% of finger flexion force, when participants pressed force sensors with the proximal phalanx in that study [41]. These studies support the idea that tMFAC stimulation inhibited MCP flexion forces contributed by the lumbrical muscles.

Our finger-pressing task demonstrated that the level of nerve inhibition differed with the magnitude of force production. Our findings indicate that a higher target force results in a greater force reduction by tMFAC stimulation. We propose that compensations within the motor system result in a smaller inhibition effect with a target force of 50% MVC, compared to a target force of 90% MVC. In the human motor system, there are abundant degrees of freedom between motor elements (e.g., body segments, muscle forces, and joints). This complex system makes it possible to obtain the same motor performance using different motor elements [42, 43]. Given that human fingers contain two types of muscles based on the origin of insertion, i.e., the intrinsic and extrinsic hand muscles, compensations between these muscles might influence motor performance during a finger-pressing task, especially when a target force is low.

Both motor and sensory nerve inhibition by tMFAC stimulation may deserve consideration as a clinical approach for reducing spasticity or pain. Spastic paralysis is defined as a motor disorder that shows increased muscle tone in a static posture and/or dynamic motor behaviors due to hyperexcitability of the stretch reflex or exaggerated cutaneous reflexes. Some earlier studies demonstrated that electrical stimulation was effective for the management of spasticity [44, 45]. In addition to spasticity, another study provided preliminary evidence on the efficacy and safety of MFAC for post-amputation pain [29]. Given that tMFAC stimulation can inhibit both motor and sensory responses, this intervention might effectively suppress spasticity and pain. However, since the current study was conducted in healthy subjects, the efficiency of tMFAC in neurologic disorders needs further investigation through clinical trials.

There are some limitations in this study. First, this is an uncontrolled study involving a small number of young healthy subjects. Although significant sensory and motor inhibition were observed in this study, the inclusion of a broader age range and/or patient population with a control group would be needed in future studies to strengthen our findings. Second, small maximum current intensity (31 mA) was used which is much smaller than the maximum current in commercial devices (100 mA [46]) and previous studies (120 mA [47–49]). Considering the intensity-dependent characteristics of tMFAC stimulation, the comfort of any tMFAC-induced sensations can be critical to achieving the desired inhibitory effects. Hence, for clinical applications, we suggest a pre-adaptation period of over 10 min of tMFAC stimulation to rapidly reduce MFAC-induced sensation and increase comfort [29]. This may result in enhanced tolerance of higher stimulation intensities, and, therefore, increased inhibition effects. Although no pre-adaptation period was used in the present experimental protocol, this period is recommended for future studies. We applied electrical simulation for a short period (5 s) because this pre-clinical study aimed to identify the technical feasibility of TENI using tMFAC and we intended to minimize the task period to reduce fatigue effects. A longer application period would be required to observe force trajectories for when a steady minimum force was reached after tMFAC stimulation. Finally, we applied MFAC at 10 kHz based on data from a previous study that used interferential current stimulation and demonstrated that, for a range of 1 Hz to 35 kHz, 10 kHz was optimal for stimulating subcutaneous tissues with minimal discomfort [30]. However, since interferential current stimulation was designed for a different technical purpose, in which low-frequency burst currents are generated for neuromuscular stimulation beneath the subcutaneous layer, further studies are required to find the optimal frequency for TENI.

## Conclusions

In this study, we developed a technique for TENI using tMFAC stimulation. We found that tMFAC stimulation significantly reduced both sensory perception and finger force production. Higher tMFAC stimulation intensities resulted in greater inhibitory effects. Motor activity was reduced immediately after stimulation. Additionally, the inhibition effect was greater when higher forces were produced. We expect that tMFAC stimulation will provide a convenient and versatile modality to inhibit undesired nerve activity, with minimal side effects. For instance, the tMFAC stimulation technique may be combined with currently used rehabilitation devices (e.g., a continuous passive motion machine, exoskeleton, or orthosis). However, the effect of tMFAC on spasticity or pain remains unknown. Further clinical studies are needed to clarify the feasibility and efficacy of TENI using tMFAC in patients with neurologic disorders.

## Abbreviations

ANOVA: Analysis of variance
FES: Functional electrical stimulation
MFAC: Medium-frequency alternating current
MSE: Mean squared error
MVC: Maximum voluntary contraction
N: Newtons
NMES: Neuromuscular electrical stimulation
TENI: Transcutaneous electrical nerve inhibition
tMFAC: Transcutaneous medium-frequency alternating current
